# Universal Minimal Model for Glucose-Insulin Relationship with the Influence of Food Dynamic

**DOI:** 10.1155/2022/8990767

**Published:** 2022-09-27

**Authors:** Kanchana Kumnungkit, Chulin Likasiri, Radom Pongvuthithum, Faifan Tantakitti

**Affiliations:** ^1^Department of Mathematics, School of Science, King Mongkut's Institute of Technology Ladkrabang, Bangkok 10520, Thailand; ^2^Research Group in Mathematics and Applied Mathematics, Department of Mathematics, Faculty of Science, Chiang Mai University, Chiang Mai 50200, Thailand; ^3^Department of Mechanical Engineering, Faculty of Engineering, Chiang Mai University, Chiang Mai 50200, Thailand

## Abstract

We propose a minimal model defining the relationship between glucose and insulin with the added influence of food intakes. The constructed model consists of a system of 3 nonlinear ordinary differential equations (ODEs). The solutions of our model for both normal and diabetic subjects are compared with a minimal model and a maximal model representing the same relationship. We found that the outputs of our model are similar to those from the minimal and maximal models for both normal and diabetic subjects; the *R*^2^ are 0.9997 and 0.9922, respectively, when compared with the minimal model, and are 0.9995 and 0.9940, respectively, when compared with the maximal model. Moreover, the relative errors between solutions are at most 0.9035% and as low as 1.488 × 10^−2^% on average when compared with the minimal model for normal subjects and at most 1.331% and as low as 0.1159% on average for diabetic subjects. The discrepancy between our model and the maximal model are at most 1.590% and 5.453% for normal and diabetic subjects, respectively, with a relative error averaging 0.2138% and 0.9002% for normal and diabetic subjects, respectively.

## 1. Introduction

Diabetes is a major health problem worldwide, with the number of people living with diabetes reaching 537 million (1 in 10 people) in 2021 and projected to rise to 783 million by 2045 [1]. Although diabetes is a chronic, not infectious, disease, diabetics are at an increased risk to develop a number of serious health problems. Some data indicate that having had a COVID-19 infection increases the risk of developing diabetes, and as one study shows [2], people with diabetes are also more likely to develop severe COVID-19 symptoms.

Diabetes is divided into 2 categories. People with type 1 diabetes produce very little to no insulin, while those with type 2 diabetes do not efficiently use the insulin their body produce. In either case, diabetes sufferers are likely to have uncontrollably high blood glucose levels. Understanding and being able to predict the effect their food intakes will have on their blood sugar levels are important for managing diabetics' food intakes, forming healthy habits, and determining effective medical interventions. Prediction of glucose level after a meal is particularly relevant since eating is a daily activity. Modeling of glucose-insulin system for oral glucose intake is complicated compared to intravenous injection because it involves additional physiological processes, including ingestion and absorption, which affect the rate of glucose appearance in the blood. This rate varies depending on the type of food and the individual's unique physiological responses to food.

Simulation models of glucose-insulin system with the added influence of glucose-containing meals have been widely developed and known to be useful for tracking various aspects of healthy and diabetic people. The series of works [3, 4] give a detailed description of such models. The model involves 6 systems of ODEs, 14 or more parameter settings, and 9 additional constraints. The clinical data from 204 healthy subjects and 14 type 2 diabetics are used to obtain the parameters of the model. In [5], a system made of 27 ODEs is used to represent the glucose and insulin submodels with parameters obtained from minimizing the errors between the model solution and clinical data for both healthy and diabetic subjects. Recently, the glucose-insulin model has been extensively improved with the combined effect of such lifestyle factors as activities, stress, meals, and medications [6]. Each submodel is given in systems of ODEs with 120 estimated parameters taken from [5].

These complicated, or maximal, models have obvious advantages as they can be verified by physiological events in the human body. However, in order to utilize the model, one has to obtain the parameters used in the model which is impractical to acquire. Moreover, the more complicated maximal models do not generally provide higher accuracy in terms of glucose-insulin dynamic [7]. Another approach to developing a simulation model for glucose-insulin dynamic, called the minimal model, seeks to simplify the model with the use of available data. By evaluating the parameters in the constructed model using data collected from studied subjects, the models can be said to represent the glucose-insulin dynamic with effects of other inputs indicated in the model. Some of the glucose-insulin minimal models in the literature can be found in [8].

Continuous glucose monitoring (CGM) uses implanted sensors to measure glucose level in the interstitial space periodically. Data from CGM are complex, and data interpretation and consequently intervention design are subjective. While models of glucose-insulin systems have been extensively studied, they are mostly based on data from oral glucose tolerance test (OGTT) and often involve many compartments such as the liver, muscle and adipose tissues, and gastrointestinal tract. Detailed physiological-based models have also been developed using data from OGTT coupled with data from radiolabeled tracers in meals and injections [7]. The GCM data that are more accessible and practical to collect are used to assess the effectiveness of the model developed in [9]. In this work, the minimal model is offered with a system of 4 ODEs. The behaviors of glucose concentration from the model are shown along with glucose data taken from one healthy and one type 2 diabetic subject.

In this work, we propose a new minimal model with the purpose to capture the glucose dynamic that is similar to the results of the minimal and maximal models. The proposed model contains only one equation for a food dynamic and two equations for glucose-insulin dynamics, as presented in the following section. The glucose dynamic behavior obtained from fitting our model is compared to the outputs from the existing models for both normal and diabetic subjects. Finally, conclusions and discussions are presented in the last section.

## 2. Model Construction and Validation

### 2.1. Food Dynamics

This work presents a novel minimal model for food dynamics. The digestion of food is modeled using a single compartment dynamic. The amount of food intake as a function of time is added into this compartment, and from this compartment, the glucose is then absorbed into the bloodstream. The food dynamics proposed here can be written as

(1) $$\begin{array}{c} \frac{dQ}{dt}=\frac{-\beta Q+\eta D\left(t\right)}{\gamma^{2}+Q^{2}}, \end{array}$$

where *Q*(*t*) is the amount of glucose in the intestine, which is readily absorbed into the bloodstream (*mg*); *D*(*t*) is the amount of ingested glucose from food intake (*mg*); *β*, *γ*, and *η* are parameters specific to the model to capture the behaviors of the glucose in the intestine, which will later be the input for the glucose-insulin dynamic model (*mg*^2^min^−1^,  *mg*, and *mg*^2^min^−1^).

Similar to other models, the rate of change of glucose depends on the amount of glucose in the intestine and the food ingested. However, the nonlinear glucose term is introduced in the denominator. This term limits the maximum rate of change of the glucose in the intestine. Compared to the food model in [9], this new approach allows us to reduce the dimension of the food model to one, and yet it can still capture the behavior of the minimal model in [9] and a much higher dimension model in [3, 4].

### 2.2. The Glucose-Insulin Dynamics

The G-I model for glucose-insulin dynamics follows from [9, 10], since it is simple, transparent, and has been widely used in literature. Along with the influence of glucose intake, the model can be described as

(2) $$\begin{array}{c} \frac{dG}{dt}=R_{0}-\left(E_{G_{0}}+S_{I}I\right)G+k_{Q}Q, \end{array}$$

(3) $$\begin{array}{c} \frac{dI}{dt}=I_{\max}\frac{G^{2}}{\alpha +G^{2}}-k_{I}I, \end{array}$$

where *G*(*t*) is the concentration of glucose in the blood stream (*mgdl*^−1^); *I*(*t*) is the concentration of insulin in the blood stream (*μUml*^−1^), where 1 *μUml*^−1^ = 6.00 *pmolL*^−1^ and 1 *mol* =5808 *g*, or 1 *U* =0.034848 *mg*; *R*_0_ is the initial rate of glucose production (*mgdl*^−1^min^−1^); *E*_*G*_0__ is the initial glucose effectiveness (min^−1^); *S*_*I*_ is the total insulin sensitivity (*mlμU*^−1^min^−1^); *k*_*Q*_ is the intestinal absorption rate (*dl*^−1^min^−1^); *I*_max_ is the total maximal insulin secretion rate in pancreatic *β*  cells (*μUml*^−1^min^−1^); *G*^2^/(*α* + *G*^2^) is the Hill function having half its maximum at *G* = *α*^1/2^; and *k*_*I*_ is the insulin clearance rate (min^−1^).

### 2.3. Model Comparison and Validation

Solutions from the complete model offered in this study, given by Equations (1)–(3), will be compared with those from both the minimal model presented in [9] and the maximal model presented in Equations 1, 3-5, 10-11, 13-19, and 23-27 from [3] along with Equation 8 from [4]. The simplified version has two-compartment dynamics. Food enters the stomach, *q*_sto_, and passes through the “gut” which is a second compartment, *q*_gut_.

(4) $$\begin{array}{c} \frac{dq_{\text{sto}}}{dt}=-k_{\text{sto}}q_{\text{sto}},\\ \frac{dq_{\text{gut}}}{dt}=k_{\text{sto}}q_{\text{sto}}-k_{\text{gut}}q_{\text{gut}}. \end{array}$$

The variable *q*_gut_(*t*) is considered to be similar to our variable *Q*(*t*).

Note that the maximal model used as a comparison reference will be given in the supplementary materials (available here) as it is rather complicated.

## 3. Results and Discussions

### 3.1. Model Analysis

The equilibrium points of the model are as follows: (1) *Q* = *βD*(*t*)/*α* for *D*(*t*) = 0, we will have *Q* = 0; (2) *G* is a positive real solution of the equation (*E*_*G*_0__ + (*S*_*I*_*I*_max_/*k*_*I*_))*G*^3^ − *R*_0_*G*^2^ + *E*_*G*_0__*αG* − *R*_0_*α* = 0; and (3) *I* = *I*_max_*G*^2^/*k*_*I*_(*α* + *G*^2^). These equilibria will be used as initial values to solve for the parameters.

### 3.2. Model Fitting

In this section, we determine the parameters in our model by minimizing the sum squared errors between the solutions of our model and those in [9] and [3, 4]. Even though fitting the model to the clinical data would allow us to determine supposedly more clinical relevant parameters, our intention in this study is to build a simpler minimal model that is still able to represent the behavior of the glucose concentration similarly to both previously published minimal and maximal models. Hence, to show this comparability, the parameters that can best fit the proposed model with those two types of model are obtained by minimizing the errors between the solutions of each pair of models. For the function *D*(*t*), we use a step function with the period of 15 minutes and the step values taken from [9] and [3, 4].

Based on the results, we found that our model is compatible with the model in [9], having the corresponding parameters provided in Table 1.

**Figure 1:**
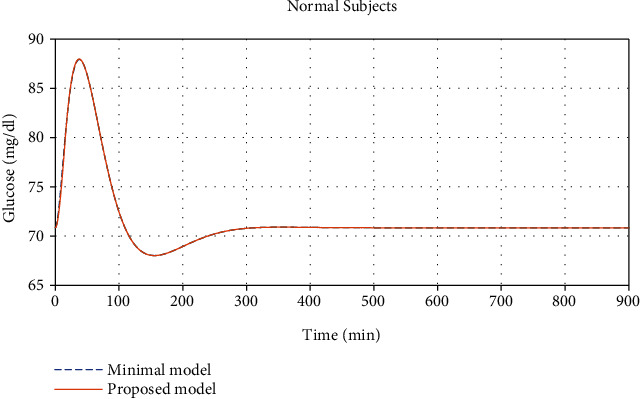
Glucose concentration comparisons between normal subjects found from the minimal model given in [9] and our proposed model (*R*^2^ = 0.9997, maximal error 0.9035%, and average error 1.488 × 10^−2^%).

**Figure 2:**
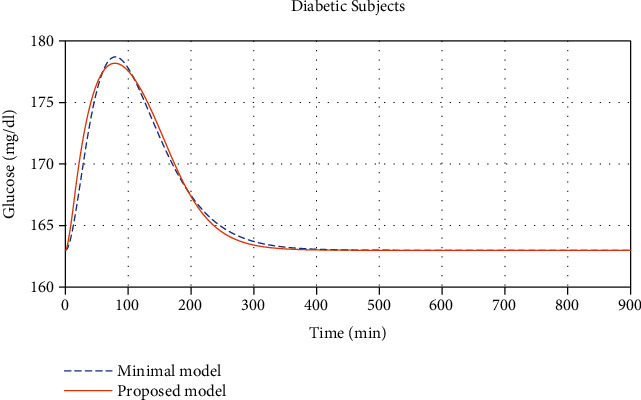
Glucose concentration comparisons between diabetic subjects found from the minimal model given in [9] and our proposed model with (*R*^2^ = 0.9922, maximal error 1.331%, and average error 0.1159%).

Comparisons between the plasma glucose concentration, *G*, (in *mg*/*dl*) found from the minimal model in [9] and from our proposed model for the normal and diabetic subjects can be seen in Figures 1 and 2, respectively.

**Figure 3:**
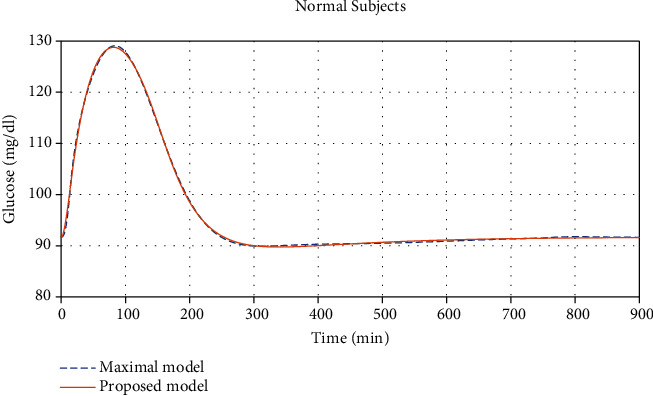
Glucose concentration comparisons between normal subjects found from the maximal model given in [4] together with [3] and our proposed model with (*R*^2^ = 0.9995, maximal error 1.59%, and average error 0.2138%).

**Figure 4:**
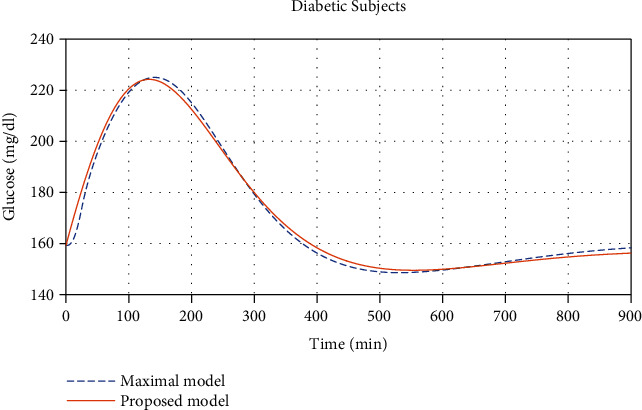
Glucose concentration comparisons between diabetic subjects found from the maximal model given in [4] together with [3] and our proposed model (*R*^2^ = 0.9940, maximal error 5.453%, and average error 0.9002%).

We then test the compatibility between our model and a maximal model for glucose and insulin relationship. We found the parameters, shown in columns 4-5 of Table 1, that best fit our solutions and the solutions obtained from the model in [3, 4], which are given in the supplementary materials. Comparisons of the solutions found from this maximal model and from our proposed model for the normal and diabetic subjects can be seen in Figures 3 and 4, respectively.

The statistics of our model show its good compatibility with those two model categories (see Table 2) with all *R*^2^ greater than 0.99. Note that both maximum and average relative errors when compared with the maximal model are higher than those with the minimal model. The solutions for normal subject cases fit better than those for diabetic subjects.

## 4. Conclusions and Discussions

There are 2 categories of glucose-insulin dynamic models: minimal models and maximal models. The model proposed in this work is a minimal model describing the relationship between glucose and insulin with the added influence of food intake. We compare our solutions with the solutions from each type of model for both normal and diabetic subjects. We found that our model behaves similarly to both the minimal and maximal models for normal subjects with the *R*^2^ lying within 0.9922-0.9997. Moreover, the errors between solutions are at most 5.453% when compared with the maximal model on diabetic subjects and as low as 0.9035% when compared with the minimal model on normal subjects. Although the *R*^2^ for the diabetic subjects are worse than that for normal subjects, values above 0.8 (in this case, 0.9922) are considered compatible statistically. One benefit of a minimal model is that the number of parameters needed to solve it is a lot smaller than those required to solve a maximal model. In this current work, the model is fitted based on the outputs of other models. Future work includes validation of our model to the clinical data. Our model with food-intake influence can be useful for those who are interested in monitoring their food intakes or meals in order to control their blood sugar levels.

## Figures and Tables

**Table 1:** Parameters in the model found through our study.

Parameters	Minimal model		Maximal model	
	Normal	Diabetics	Normal	Diabetics
*β*	6.050	1.778	3.252	0.3353
*γ*	12.86	7.089	7.851	135.4
*η*	4.086	4.641	11.98	1.337
*R*_0_	2.1	2.5	1.812	1.046
*E*_*G*_0__	1 × 10^−3^	2.5 × 10^−3^	1.943 × 10^−2^	2.212 × 10^−8^
*S*_*I*_	3.06 × 10^−3^	1.14 × 10^−3^	1.340 × 10^−4^	1.290 × 10^−5^
*α*	1 × 10^4^	1 × 10^4^	9193.4	2.903 × 10^5^
*I*_max_	0.28	0.93	3.117 × 10^−2^	24.31
*k*_*I*_	0.01	0.06	6.007 × 10^−3^	3.638 × 10^−3^
*k*_*Q*_	0.098	0.026	5.880 × 10^−2^	2.572 × 10^−8^

**Table 2:** Statistics of the proposed model compared with minimal and maximal models.

	Minimal model		Maximal model	
	Normal	Diabetics	Normal	Diabetics
*R* ^2^	0.9997	0.9922	0.9995	0.9940
Average error (%)	1.488 × 10^−2^	0.1159	0.2138	0.9002
Maximum error (%)	0.9035	1.331	1.590	5.453

## Data Availability

There is no collected data in this study. All data are obtained from simulation which can be found from parameters given in the manuscript.

## Supplementary Materials

Supplementary Materials Supplementary file 1 contains the maximal model from [3, 4] that is used for comparison.
