# Supplementary material for: Universal Minimal Model for Glucose-Insulin Relationship with the Influence of Food Dynamic
Source: Comput Math Methods Med. 2022 Sep 27;2022:8990767. doi: 10.1155/2022/8990767 (PMC9532137; doi:10.1155/2022/8990767)
Supplement: Supplementary Materials — Supplementary file 1 contains the maximal model from [3, 4] that is used for comparison. [file 8990767.f1.docx]

**Supplementary materials**

**The maximal model used as a comparison reference from reference [3-4] is as follow:**

**Glucose subsystem**

$\frac{dG_{p}}{dt}=EGP\left( t \right)+Ra\left( t \right)-U_{ii}\left( t \right)-E\left( t \right)-k_{1}\cdot G_{p}\left( t \right)+k_{2}\cdot G_{t}\left( t \right), G_{p}\left( 0 \right){=G}_{pb}$

$\frac{dG_{t}}{dt}=-U_{id}\left( t \right)+k_{1}\cdot G_{p}\left( t \right)-k_{2}\cdot G_{t}\left( t \right), G_{t}\left( 0 \right){=G}_{tb}$

$G\left( t \right)=\frac{G_{p}}{V_{G}} G\left( 0 \right)=G_{b}$

**Insulin subsystem**

$\frac{dI_{l}}{dt}=-{(m}_{1}+m_{3}\left( t \right))\cdot I_{l}(t)+m_{2}I_{p}\left( t \right)+ S(t), I_{l}\left( 0 \right){=I}_{lb}$

$\frac{dI_{p}}{dt}=-{(m}_{2}+m_{4})\cdot I_{p}(t)+m_{1}I_{l}\left( t \right), I_{p}\left( 0 \right){=I}_{pb}$

$I\left( t \right)=\frac{I_{p}}{V_{l}} I\left( 0 \right)=I_{b}$

$HE\left( t \right)=-m_{5}\cdot S\left( t \right)+m_{0} HE\left( 0 \right)={HE}_{b}$

$m_{3}\left( t \right)=\frac{HE(t)\cdot m_{1}}{1-HE(t)}$

**Endogenous glucose production**

$EGP\left( t \right)=k_{p1}-k_{p2}\cdot G_{p}\left( t \right)-k_{p3}\cdot I_{d}\left( t \right)-k_{p4}\cdot I_{p0}\left( t \right)$

$EGP\left( 0 \right)={EGP}_{b}$

$\frac{dI_{1}}{dt}=-k_{i}\cdot\left[ I_{l}\left( t \right)-I(t) \right], I_{1}\left( 0 \right){=I}_{b}$

$\frac{dI_{d}}{dt}=-k_{i}\cdot\left[ I_{d}\left( t \right)-I_{1}(t) \right], I_{d}\left( 0 \right){=I}_{b}$

**Glucose rate of appearance**

$\frac{dQ_{sto}}{dt}=Q_{sto1}\left( t \right)+Q_{sto2}\left( t \right), Q_{sto}\left( 0 \right)=0$

$\frac{dQ_{sto1}}{dt}=-k_{gri}\cdot Q_{sto1}\left( t \right)+D\cdot d\left( t \right), Q_{sto1}\left( 0 \right)=0$

$\frac{dQ_{sto2}}{dt}=-k_{empt}(Q_{sto})\cdot Q_{sto2}\left( t \right)+k_{gri}\cdot Q_{sto1}\left( t \right), Q_{sto2}\left( 0 \right)=0$

$Q_{gut}=-k_{abs}\cdot Q_{gut}\left( t \right)+k_{empt}(Q_{sto})\cdot Q_{sto2}\left( t \right), Q_{gut}\left( 0 \right)=0$

$Ra\left( t \right)=\frac{f\cdot k_{abs}\cdot Q_{gut}(t)}{BW}$

$k_{empt}\left( Q_{sto} \right)=k_{min}+\frac{k_{max}{-k}_{min}}{2}\cdot\left[ \text{tanh}\left( \alpha(q_{sto}-b\cdot D) \right)-\text{tanh}\left( \beta\left( q_{sto}-c\cdot D \right) \right)+2 \right]$

$q_{sto}\left( t \right)=q_{sto1}\left( t \right)+q_{sto2}(t)$

**Glucose Utilization**

$U_{ii}\left( t \right)=F_{cns}$

$U_{id}\left( t \right)=\frac{V_{m}(X\left( t \right))\cdot G_{t}(t)}{K_{m}(X\left( t \right)+G_{t}\left( t \right))}$

$V_{m}\left( X\left( t \right) \right)=V_{m0}+V_{mx}\cdot X(t)$

$K_{m}\left( X\left( t \right) \right)=K_{m0}+K_{mx}\cdot X(t)$

$\frac{dX}{dt}=-p_{2U}\cdot X\left( t \right)+p_{2U}\left[ I\left( t \right)-I_{b} \right], X\left( 0 \right)=0$

$U\left( t \right)=U_{ii}\left( t \right)+U_{id}(t)$

**Insulin Secretion**

$S\left( t \right)=\gamma\cdot I_{po}(t)$

$\frac{dI_{po}}{dt}=-\gamma\cdot I_{po}\left( t \right)+S_{po}\left( t \right), I_{po}\left( 0 \right)=I_{pob}$

$S_{po}\left( t \right)=\left\{ \begin{matrix} Y\left( t \right)+K\cdot\frac{dG}{dt}+S_{b} \text{for}\frac{dG}{dt}>0 \\ Y\left( t \right)+S_{b} \text{for}\frac{dG}{dt}\leq0 \end{matrix} \right.$

$\frac{dY}{dt}=\left\{ \begin{matrix} -\alpha\cdot\left[ Y\left( t \right)-\beta\cdot\left( G\left( t \right)-h \right) \right] \text{if} \beta\cdot\left( G\left( t \right)-h \right)\geq-S_{b} \\ -\alpha\cdot Y\left( t \right)-\alpha{\cdot S}_{b} \text{ if} \beta\cdot\left( G\left( t \right)-h \right)<-S_{b} \end{matrix} \right. Y\left( 0 \right)=0$

**Glucose Renal Excretion**

$E\left( t \right)=\left\{ \begin{matrix} k_{e1}\cdot\left[ G_{p}\left( t \right)-k_{e2} \right] \text{if }G_{p}\left( t \right)>k_{e2} \\ 0 \text{if }G_{p}\left( t \right)\leq k_{e2} \end{matrix} \right.$

The details of each equation and all parameter values are given in [3] and [4].
